# The socio-economic burden of snakebite in Sri Lanka

**DOI:** 10.1371/journal.pntd.0005647

**Published:** 2017-07-06

**Authors:** Anuradhani Kasturiratne, Arunasalam Pathmeswaran, A. Rajitha Wickremasinghe, Shaluka F. Jayamanne, Andrew Dawson, Geoff K. Isbister, Hithanadura Janaka de Silva, David G. Lalloo

**Affiliations:** 1 Faculty of Medicine, University of Kelaniya, Ragama, Sri Lanka; 2 South Asian Clinical Toxicology Research Collaboration (SACTRC), Faculty of Medicine, University of Peradeniya, Peradeniya, Sri Lanka; 3 Clinical Toxicology Research Group, University of Newcastle, Callaghan, Australia; 4 Liverpool School of Tropical Medicine, Liverpool, United Kingdom; Universidad de Costa Rica, COSTA RICA

## Abstract

**Background:**

Snakebite is a major problem affecting the rural poor in many of the poorest countries in the tropics. However, the scale of the socio-economic burden has rarely been studied. We undertook a comprehensive assessment of the burden in Sri Lanka.

**Methods:**

Data from a representative nation-wide community based household survey were used to estimate the number of bites and deaths nationally, and household and out of pocket costs were derived from household questionnaires. Health system costs were obtained from hospital cost accounting systems and estimates of antivenom usage. DALYs lost to snakebite were estimated using standard approaches using disability weights for poisoning.

**Findings:**

79% of victims suffered economic loss following a snakebite with a median out of pocket expenditure of $11.82 (IQR 2–28.57) and a median estimated loss of income of $28.57 and $33.21 for those in employment or self-employment, respectively. Family members also lost income to help care for patients. Estimated health system costs for Sri Lanka were $ 10,260,652 annually. The annual estimated total number of DALYS was 11,101 to 15,076 per year for envenoming following snakebite.

**Interpretation:**

Snakebite places a considerable economic burden on the households of victims in Sri Lanka, despite a health system which is accessible and free at the point of care. The disability burden is also considerable, similar to that of meningitis or dengue, although the relatively low case fatality rate and limited physical sequelae following bites by Sri Lankan snakes means that this burden may be less than in countries on the African continent.

## Author summary

Snakebite predominantly affects poor people in the rural tropics. The effect that snakebite has on these populations, both economically and in terms of death and disability, is poorly understood. We used data from a national household survey of snakebite in Sri Lanka to estimate the burden of death and disability and to calculate the financial cost of a snakebite episode for the Sri Lankan health system and for Sri Lankan households. We found that the burden of snakebite was considerable, similar to that of common diseases like meningitis or dengue and that treating snakebite cost the Sri Lankan government over $10 million each year. Despite health care being free in Sri Lanka, almost 80% of households experienced additional costs and loss of income following a snakebite; such costs are disastrous for poor rural workers.

## Introduction

Snakebite is a major public health problem in rural communities in Asia, Africa and Latin America [1]. The problem has been extensively studied from a bio-medical perspective but rarely from a socio-economic viewpoint. However, the negative social and economic consequences of snakebite are likely to be considerable. Firstly, snakebite is a problem of tropical low and middle income countries that are already facing the considerable dual burden of communicable and non-communicable diseases; snakebite mortality is strongly associated with low per capita Gross Domestic Product and a low Human Development Index [2]. In such settings, the ill-health associated with snakebite can exert a considerable burden on the economic development of these countries. Secondly, the victims are usually economically productive, young individuals in these communities whose future productive lifespan can be negatively affected by snakebite. Thirdly, most of those affected are rural daily wage earners employed in the informal sector that include farming and other labour intensive occupations, for whom snakebite results in a considerable opportunity cost for being away from work.

Very few studies have attempted to formally investigate the socio-economic burden associated with snakebite. A household survey in rural India demonstrated a significant reduction in medium and long-term family income due to snakebite, in addition to the immediate costs of a bite [3]. A West African study estimated years of life lived with disability (YLD) due to limb amputations resulting from snakebite using DALYs for 16 countries in the region [4]. Disability-adjusted-life years (DALYs) is a widely used metric for estimating disease burden based on strong economic and ethical principles [5]. The Global Burden of Disease (GBD) Study used DALYs successfully to describe disability and the burden of important diseases for the period 1990–2020 [6]. Since then, the method of GBD estimation has been improved in repeated attempts to estimate the global disease burden [7]. National estimates of the overall social and economic impact of snakebite have not been attempted for any country to date. This paper estimates the economic cost and disease burden of snakebite for Sri Lanka using data from a recent country-wide community based survey [8].

## Methods

### Ethics statement

The study was approved by the Ethics Review Committee of the Faculty of Medicine, University of Kelaniya. Permission for conducting the study was obtained from District and Divisional level public administrators before data collection. Grama Niladharis of the sampled GN divisions were informed about the study through the public administration system. Written informed consent was obtained from the participants before data collection. The information sheet was read out to illiterate patients in the presence of a family member and a witnessed thumb print was used to signify consent.

A country-wide community based cross sectional survey was conducted between August 2012 and June 2013 [8]. The survey was designed to sample approximately 1% of the population of Sri Lanka distributed equally among its nine provinces. A Grama Niladhari (GN) division (the smallest administrative unit in the country) was defined as a cluster for data collection, and 125 clusters were allocated to each of the 9 provinces. Within each province the number of clusters was divided among the districts in proportion to each districts’ population. The clusters were selected using simple random sampling from the list of GN divisions available at the Department of Census and Statistics, Sri Lanka. 40 households were sampled consecutively from the randomly selected starting point in each cluster.

Information related to all residents of the sampled households was obtained by trained data collectors using an interviewer administered questionnaire. They were assisted by local field volunteers recruited from within the cluster. The respondent of each household was either the head of the household or a responsible adult present in the house. A two part structured questionnaire was used for data collection. The questionnaire was translated into Sinhala and Tamil and was pre-tested in a GN division within each province which was not selected for the study. Based on the findings of the pretesting, the questionnaire was fine-tuned prior to use.

In the first phase of data collection, the research assistant screened the households for snakebite within the previous 12 months and obtained socio-demographic data from the households. In the second phase of data collection, instruments were administered to the households which had reported snakebites within the previous 12 months in order to obtain details of the bite, clinical manifestations of envenoming, residual disability and deaths due to snakebite. Detailed information on the household costs of snakebite was recorded. Only systemic symptoms or signs were considered to reflect envenoming.

Data were double entered into databases created in Epidata software. Discrepancies were corrected by referring to the original data sheets. Data analysis was performed in SPSS version 22.

### Estimating the cost of snakebite

The median out-of-pocket cost of different cost elements were estimated based on the data reported by the victims or a household member for a number of different out of pocket costs. Victims or household members were also asked to estimate the number of days lost off work due to the snakebite and the amount of wages lost by the victim and the household due to the snakebite. The total sum spent by patients for a particular cost item and the proportion of patients that incurred that cost was applied to the estimated national incidence of snakebite to estimate the total annual out-of-pocket cost of snakebite for the entire country. The income lost by the patient or family members and the proportion of patients who lost income in different ways were applied to the estimated national incidence of snakebite to arrive at the total annual lost income due to snakebite for the entire country. The health system cost of snakebite was estimated based on cost data obtained from the cost accounting system maintained at the Teaching Hospital, Kurunegala, Sri Lanka. The average cost of a patient day in the medical ward excluding drug costs was obtained from this database. This cost included the cost of nursing and medical care, investigations and the hotel costs of maintaining a patient in a medical ward. The cost of a patient day amounted to LKR 3214.00 (USD 22.96). Ancillary treatments are rarely used in Sri Lanka and were therefore not included. The cost of a vial of anti-venom was obtained from the price list issued by the Medical Supplies Division, Ministry of Health, Sri Lanka [9]. The number of anti-venom vials used was estimated based on the national guidelines for management of snakebite and the assumption that the 30%, 65% and 5% of envenomed patients received respectively, 10, 20 and 30 vials of anti-venom during the management of envenoming. This assumption was based on the consensus arrived among five specialist physicians experienced in managing snakebite in different parts of the country. The median duration of hospitalization for a snakebite with and without envenoming was estimated based on the data reported by the households of victims. The costs were extrapolated for the country using the estimated national incidence of snakebite from the nationwide survey.

### Estimating disability-adjusted life years

DALYs were calculated using the following formula: DALY = YLD + YLL. The template developed by the World Health Organization [10] was used for estimation of DALYs. Population data from the 2012 national census were obtained from the Department of Census and Statistics, Sri Lanka ([11]. For envenoming, the disability weight used for the higher estimate was 0.6 which is the accepted disability weight used for poisoning in the original GBD study [6]. We used a disability weight of 0.163 for the lower estimate based on the disability weight used for poisoning in the 2013 GBD study. In using the disability weight for poisoning, we have assumed that snakebite envenoming and poisoning are comparable in terms of the associated disability. The duration of an episode of snakebite with envenoming was considered to be 0.3 years. For snakebites without envenoming disability weights of 0.006 (lower estimate) and 0.108 (higher estimate) were used. These are the weights used for open wounds in the GBD studies [6,12]. The duration of illness for snakebite without envenoming was considered to be 0.04 years. The standard discount rate used was 0.03. Beta and constant values used for standard age weighting were 0.04 and 0.1658.

## Results

The total sample was 165,665 individuals living in 44,136 households approximately equally distributed among the nine provinces of the country. The survey reported 695 snakebites and five deaths. Envenoming had been observed in 323 bite victims. Residual physical disability following the bite was reported by 59 (8.5%) victims. For the entire country, the extrapolated number of snakebites, envenoming and deaths were 80,277, 30,458, and 429, respectively [8].

### Household cost of snakebite

551 (79.3%) victims incurred an economic loss following a bite and 550 (79.1%) victims incurred out-of-pocket expenditure for healthcare. The median total out-of-pocket expenditure per snakebite episode (envenomed and non-envenomed) was USD 11.82 (Inter-quartile range 5–28.57). Details of out-of-pocket expenditure are given in Table 1 and include travel, food, costs of keeping carers with the victim during the hospital stay, fees for laboratory investigation, purchase of pharmaceuticals and medical products that were not available in the hospital and other unspecified direct costs. In addition, a cost was often incurred for religious and cultural rituals which were organized for 138 (19.9%) victims necessitating expenditure for 101 (14.5%) families. The median cost of conducting these activities was USD 7.14 (Inter quartile range 3.57–14.29). The annual estimated national direct out-of–pocket expenditure for snakebite was USD 1,981,699.

**Table 1:** Out-of-pocket expenditure following snakebite.

Cost item	Number (%) experiencing cost (National survey)	Estimated number of patients incurring the cost nationally	Mean expenditure (SD) for those experiencing cost (USD)*	Median cost (IQR) USD*	Total cost for survey patients LKR (USD)*	Estimated national cost (LKR)*	Estimated national cost (USD)*
**Transport of victim**	489 (70.4)	56,515	7.82 (14.22)	3.93 (2.2–7.1)	535,110 (3,822)	61,844,052	441,743
**Visits by family**	288 (41.4)	33,235	16.89 (94.80)	7.14 (3.6–14.3)	680,858 (4863.27)	78,570,540	561,218
**Help and carers**	78 (11.2)	8,991	11.17 (14.11)	7.14 (3.6–11.6)	121,950 (871.07)	14,057,083	100,408
**Food**	225 (32.4)	26,010	10.15 (13.86)	7.14 (3.6–14.3)	319,575 (2282.68)	36,942,870	263,878
**Pharmaceuticals/ Medicines**	126 (18.1)	14,530	15.23 (65.30)	5.71 (2.5–8.9)	268.705 (1919.32)	30,986,378	221,331
**Laboratory Investigations**	31 (4.5)	3,612	21.38 (36.58)	8.57 (4.6–13.4)	92,780 (662.71)	10,810,366	77,217
**Medical Products**	12 (1.7)	1,365	4.55 (8.81)	1.96 (0.7–3.6)	7,640 (5,457)	869,050.00	6,208
**Unspecified costs**	65 (9.4)	7,546	17.50 (95.23)	3.57 (1.4–7.9)	159,220 (1137.29)	18,484,217	132,030
							
**Religious/ cultural rituals**	101 (14.5)	11640	17.50 (95.23)	7.14 (3.6–14.3)	215,825 (1541.61)	24,873,297	177,666
**Total**						**277,437,853**	**1,981,699**

*1 USD = 140 Sri Lankan Rupees (LKR)

442 of the victims (63.6%) were employed, but only 134 (19.3%) reported that they had to stop work temporarily due to the bite. The median total income lost due to the bite by these victims was USD 28.57 (Inter-quartile range 17.14–56.07) (Table 2). Self-employed victims (n = 158, 22.7%) lost a median income of USD 33.21 (Inter-quartile range 17.86–54.46) either due to lost work or costs of a replacement. National annual estimated lost income was USD 910,259 and USD 844,142 for employed and self-employed victims respectively. At least one family member of 103 (14.8%) bite victims had lost at least one workday due to the bite. The median economic loss by these family members due to the bite was USD 28.57 (Inter-quartile range 14.29–81.07). This amounted to an annual estimated lost income of USD 101,037 for family members of victims. Overall, the estimated annual lost income amounted to a total of USD 1,855,438, meaning that the total annual economic burden on households nationally was USD 3,837,137.

**Table 2:** Indirect household costs of snakebite.

Cost item	Number (%) experiencing cost (National survey)	Estimated number of patients incurring the cost nationally	Mean expenditure (SD) for those experiencing cost (USD)*	Median cost (IQR) USD*	Total cost for survey patients LKR (USD)*	Estimated national cost (LKR)*	Estimated national cost (USD)*
**Employed victims**	125 (18.0)	14,450	62.99 (96.01)	28.57 (17.1–56.1)	1,102,390 (7874.2)	127,436,284	910,259
**Self- employed**	158(22.7)	17,661	47.80 (80.66)	33.21 (17.9–54.5)	1,023,810 (7312.9)	118,179,794	844,142
**Relatives**	36 (5.2)	4,174	24.21 (12.07)	28.57 (14.3–81.1)	122,000 (871.4)	14,145,222	101,037
**Total**						**259,761,300**	**1,855,438**

*1 USD = 140 Sri Lankan Rupees (LKR)

### Health system cost of snakebite

The estimated annual health system cost of snakebite management was USD 10,260,651.53 (Table 3). This comprised approximately USD 6.3 million for the snakebite anti-venom and USD 3.9 million for the hospital management of patients (Table 2). Combining household and health system costs meant that the estimated total annual economic burden of snakebite was USD 14,097,789.

**Table 3:** Estimated annual health system cost of snakebite in Sri Lanka.

Clinical Presentation	Number of Patients	Duration of Hospitalization (Days)	Total ward cost without AVS (USD)	Cost of AVS (USD)	Total cost (USD)
Without envenoming	49 819	1	1,143,701.90	-	1,143,701.90
With envenoming	30 458	4	2 796 914.63	6 320,035.00	9,116,949.63
	80 277	-	3 940 616.53	6,320,035.00	10,260,651.53

### Disability burden of snakebite

The total YLL due to snakebite was 4,765 for males and 4,853 for females. Total YLD ranged from 859–3,161 for males and 624–2,296 for females. The annual estimated total number of DALYs for envenoming and death due to snakebite ranged from a lower estimate of 11, 101 to an upper estimate of 15,076 per year. This comprised 5,624–7,927 DALYs for males and 5477–7,150 DALYs for females, equating to 0.5–0.7 DALYs per 1000 population for snakebite envenoming (Tables 4 and 5). The total estimated DALYs due to snakebites without envenoming ranged from 20–500 per year (S1 and S2 Tables).

**Table 4:** Annual disease burden of snakebite envenoming in Sri Lanka (lower estimate)*.

	*Males*			*Females*			*Total*		
	Population	DALYs	DALYs per 1,000	Population	DALYs	DALYs per 1,000	Population	DALYs	DALYs per 1,000
***Age***									
**0–4**	879,223	34	0	864,639	99	0.1	1,743,862	133	0.1
**5–14**	1,711,177	346	0.2	1,676,627	278	0.2	3,387,804	624	0.2
**15–29**	2,305,753	1,349	0.6	2,424,227	1,025	0.4	4,729,980	2,374	0.5
**30–44**	2,144,526	1,827	0.9	2,263,175	1,580	0.7	4,407,701	3,407	0.8
**45–59**	1,700,304	1,645	1	1,869,215	2,019	1.1	3,569,519	3,664	1
**60–69**	709,192	326	0.5	842,007	384	0.5	1,551,199	710	0.5
**70–79**	298,235	97	0.3	397,365	92	0.2	695,600	189	0.3
**80+**	108,224			165,550			273,774		
**Total**	**9,856,634**	**5,624**	**0.6**	**10,502,805**	**5,477**	**0.5**	**20,359,439**	**11,101**	**0.5**

*Estimated using a disability weight of 0.163

**Table 5:** Annual disease burden of snakebite envenoming in Sri Lanka (higher estimate)**.

	Males			Female			Total		
*Age*	Population	DALYs	DALYs per 100	Population	DALYs	DALYs per 100	Population	DALYs	DALYs per 100
**0–4**	879,223	45	0.1	864,639	119	0.1	1,743,862	165	0.1
**5–14**	1,711,177	457	0.3	1,676,627	345	0.2	3,387,804	802	0.2
**15–29**	2,305,753	1,812	0.8	2,424,227	1,287	0.5	4,729,980	3,099	0.7
**30–44**	2,144,526	2,521	1.2	2,263,175	2,025	0.9	4,407,701	4,546	1.0
**45–59**	1,700,304	2,403	1.4	1,869,215	2,690	1.4	3,569,519	5,093	1.4
**60–69**	709,192	517	0.7	842,007	541	0.6	1,551,199	1,058	0.7
**70–79**	298,235	171	0.6	397,365	142	0.4	695,600	314	0.5
**80+**	108,224	-	-	165,550	-	-	273,774	-	-
**Total**	**9,856,634**	**7927**	**0.8**	**10,502,805**	**7,150**	**0.7**	**20,359,439**	**15,076**	**0.7**

**Estimated using a disability weight of 0.6.

## Discussion

Snakebite is a major neglected tropical disease. The relative lack of data on the burden of snakebite demonstrates the lack of attention to this condition which is confined to poor rural areas of tropical, low and middle income countries [2]. We have estimated the societal and economic burden of snakebite using nationally representative data generated from a large scale community survey conducted in Sri Lanka. We estimate that snakebite is responsible for up to approximately 15,000 DALYs per year. This is three times greater than the estimate from the Institute for Health Metrics and Evaluation for Sri Lanka and would suggest that the burden of snakebite in Sri Lanka is equivalent to that of meningitis and greater than that of dengue [13].

Given the numbers of snakebites in Sri Lanka, the estimated DALYs due to snakebite in this paper are relatively modest compared to those estimated from West Africa [4]. The availability of antivenom and supportive facilities in an accessible and free health system has led to a reduction in snakebite mortality over the last two decades in Sri Lanka to the point where case fatality rates are now only 1.5% in envenomed patients [8]. This situation contrasts with the limited access to healthcare facilities and poor availability of antivenom in many parts of Africa, where snakebite occurs in the poorest rural populations [2]. The current crisis in antivenom supply for Africa means that many patients die because they simply cannot be treated [14]. In addition, a major contributor to the burden calculation for West Africa was the disability that results from severe tissue damage and consequent amputation, an outcome that is uncommon following envenoming by venomous species in Sri Lanka [4,15].

High quality facilities and emergency care coupled with adequate supplies of anti-venom lead to the good outcomes following snakebite in Sri Lanka, but means the estimated health system costs of managing snakebite are considerable at over USD 10 million and account for 0.7% of total government health expenditure [16]. It is unlikely that these costs will reduce in the near future as there is no indication that the high incidence of bites is declining and improving health seeking behaviour means that western standard healthcare facilities are increasingly likely to be used.

Even more concerning is the economic burden that snakebite places on victims and their households. Few studies have previously attempted to estimate the effect of snakebite on this although one Tamil Nadu study demonstrated the ongoing adverse household consequences of expenditure on snakebite, requiring loans and selling of household assets to pay for treatment costs and the economic burden of snakebite upon households has also been noted in Bangladesh [3,17]. Our research demonstrated the substantial household costs of an episode of snakebite from both out of pocket costs and lost income. Some workers in the formal employment may be compensated by paid sick-leave, but only a few victims of snakebite have formal employment. The median household cost of an episode of envenoming was $12 and around a fifth of patients reported losing income of approximately $30. To put this in context, the annual per capita expenditure on health in 2014 was $127 [18] and the mean per capita income in the rural areas was $74 per month [19]. The national household economic burden of snakebite amounted to USD 3.8 million and it is highly likely in Sri Lanka that snakebite drives the same catastrophic costs for the poor as many other diseases [19,20].

There are clearly limitations to this study. Many of the estimates depend on assumptions about the duration of disability and recall of individuals about the costs that they incurred. There were challenges in the estimation of disability burden as there are no accepted disability weights for snakebite and so those for poisoning were used for envenomed individuals and assumptions regarding wounds were made for non-envenomed. Despite this, to our knowledge, this is the first ever comprehensive estimation of a national socio-economic burden from snakebite. Our results demonstrate the extent of this burden in Sri Lanka and highlights the considerable physical and economic impact of this disease, both upon the country and upon the lives of poor rural workers.

## Supporting information

S1 Dataset DALY dataset. (XLS)

S1 Table Total annual DALYs attributable to snakebite in Sri Lanka (lower estimate). (DOCX)

S2 Table Total annual DALYs attributable to snakebite in Sri Lanka (higher estimate). (DOCX)
