# Supplementary material for: The socio-economic burden of snakebite in Sri Lanka
Source: PLoS Negl Trop Dis. 2017 Jul 6;11(7):e0005647. doi: 10.1371/journal.pntd.0005647 (PMC5500261; doi:10.1371/journal.pntd.0005647)
Supplement: S1 Table — (DOCX) [file pntd.0005647.s002.docx]

Supplementary Table 1: Total annual DALYs attributable to snakebite in Sri Lanka (lower estimate)

|  | ***Males*** | | | ***Females*** | | | ***Total*** | | |
| --- | --- | --- | --- | --- | --- | --- | --- | --- | --- |
|  | **Population** | **DALYs** | **DALYs per** | **Population** | **DALYs** | **DALYs per** | **Population** | **DALYs** | **DALYs per** |
|  |  |  | **1,000** |  |  | **1,000** |  |  | **1,000** |
|  |  |  |  |  |  |  |  |  |  |
| ***Age*** |  |  |  |  |  |  |  |  |  |
| **0-4** | 879,223 | 34 | 0 | 864,639 | 99 | 0.1 | 1,743,862 | 133 | 0.1 |
| **5-14** | 1,711,177 | 347 | 0.2 | 1,676,627 | 278 | 0.2 | 3,387,804 | 625 | 0.2 |
| **15-29** | 2,305,753 | 1,351 | 0.6 | 2,424,227 | 1,027 | 0.4 | 4,729,980 | 2,378 | 0.5 |
| **30-44** | 2,144,526 | 1,831 | 0.9 | 2,263,175 | 1,582 | 0.7 | 4,407,701 | 3,413 | 0.8 |
| **45-59** | 1,700,304 | 1,649 | 1 | 1,869,215 | 2,022 | 1.1 | 3,569,519 | 3,671 | 1 |
| **60-69** | 709,192 | 327 | 0.5 | 842,007 | 385 | 0.5 | 1,551,199 | 712 | 0.5 |
| **70-79** | 298,235 | 97 | 0.3 | 397,365 | 92 | 0.2 | 695,600 | 189 | 0.3 |
| **80+** | 108,224 | - | - | 165,550 | - | - | 273,774 | - | - |
| **Total** | **9,856,634** | **5,636** | **0.6** | **10,502,805** | **5,485** | **0.5** | **20,359,439** | **11,121** | **0.5** |
