# Supplementary material for: The socio-economic burden of snakebite in Sri Lanka
Source: PLoS Negl Trop Dis. 2017 Jul 6;11(7):e0005647. doi: 10.1371/journal.pntd.0005647 (PMC5500261; doi:10.1371/journal.pntd.0005647)
Supplement: S2 Table — (DOCX) [file pntd.0005647.s003.docx]

Supplementary Table 2: Total annual DALYs attributable to snakebite in Sri Lanka (higher estimate)

|  | ***Males*** | | | ***Females*** | | | ***Total*** | | |
| --- | --- | --- | --- | --- | --- | --- | --- | --- | --- |
|  | **Population** | **DALYs** | **DALYs per** | **Population** | **DALYs** | **DALYs per** | **Population** | **DALYs** | **DALYs per** |
|  |  |  | **1,000** |  |  | **1,000** |  |  | **1,000** |
|  |  |  |  |  |  |  |  |  |  |
| ***Age*** |  |  |  |  |  |  |  |  |  |
| **0-4** | 879,223 | 47 | 0.1 | 864,639 | 122 | 0.1 | 1,743,862 | 169 | 0.1 |
| **5-14** | 1,711,177 | 472 | 0.3 | 1,676,627 | 352 | 0.2 | 3,387,804 | 824 | 0.2 |
| **15-29** | 2,305,753 | 1,847 | 0.8 | 2,424,227 | 1,332 | 0.5 | 4,729,980 | 3,179 | 0.7 |
| **30-44** | 2,144,526 | 2,618 | 1.2 | 2,263,175 | 2,073 | 0.9 | 4,407,701 | 4,691 | 1.1 |
| **45-59** | 1,700,304 | 2,515 | 1.5 | 1,869,215 | 2,750 | 1.5 | 3,569,519 | 5,264 | 1.5 |
| **60-69** | 709,192 | 552 | 0.8 | 842,007 | 567 | 0.7 | 1,551,199 | 1,118 | 0.7 |
| **70-79** | 298,235 | 178 | 0.6 | 397,365 | 154 | 0.4 | 695,600 | 332 | 0.5 |
| **80+** | 108,224 | - | - | 165,550 | - | - | 273,774 | - | - |
| **Total** | **9,856,634** | **8,229** | **0.8** | **10,502,805** | **7,348** | **0.7** | **20,359,439** | **15,576** | **0.8** |
